# Coarse-Grained Pruning of Neural Network Models Based on Blocky Sparse Structure

**DOI:** 10.3390/e23081042

**Published:** 2021-08-13

**Authors:** Lan Huang, Jia Zeng, Shiqi Sun, Wencong Wang, Yan Wang, Kangping Wang

**Affiliations:** 1 College of Computer Science and Technology, Jilin University, Changchun 130012, China; huanglan@jlu.edu.cn (L.H.); zengjia19@mails.jlu.edu.cn (J.Z.); sunsq20@mails.jlu.edu.cn (S.S.); wangwc18@mails.jlu.edu.cn (W.W.); 2 Key Laboratory of Symbolic Computation and Knowledge Engineering of the Ministry of Education, Jilin University, Changchun 130012, China

**Keywords:** neural network compression, block pruning, sparse matrix computation

## Abstract

Deep neural networks may achieve excellent performance in many research fields. However, many deep neural network models are over-parameterized. The computation of weight matrices often consumes a lot of time, which requires plenty of computing resources. In order to solve these problems, a novel block-based division method and a special coarse-grained block pruning strategy are proposed in this paper to simplify and compress the fully connected structure, and the pruned weight matrices with a blocky structure are then stored in the format of Block Sparse Row (BSR) to accelerate the calculation of the weight matrices. First, the weight matrices are divided into square sub-blocks based on spatial aggregation. Second, a coarse-grained block pruning procedure is utilized to scale down the model parameters. Finally, the BSR storage format, which is much more friendly to block sparse matrix storage and computation, is employed to store these pruned dense weight blocks to speed up the calculation. In the following experiments on MNIST and Fashion-MNIST datasets, the trend of accuracies with different pruning granularities and different sparsity is explored in order to analyze our method. The experimental results show that our coarse-grained block pruning method can compress the network and can reduce the computational cost without greatly degrading the classification accuracy. The experiment on the CIFAR-10 dataset shows that our block pruning strategy can combine well with the convolutional networks.

## 1. Introduction

Deep neural network architectures are becoming more complex, and the number of parameters is also increasing sharply [1,2]. Such a large number of parameters and operations cost a lot of storage and computing resources. In order to reduce the number of parameters and to accelerate the computational process, many methods for neural network compression and pruning have been proposed, such as low-rank factorization [3], knowledge distillation [4], and weight sharing and connection pruning [5], etc. For traditional connection pruning strategies, many fine-grained pruning methods only remove individual connections [6,7]. These pruning processes allow the network to reach a higher sparsity level but lead to irregular computing patterns. The distribution of zero elements is scattered in the irregular weight matrix such that the unique computational advantages of zero in the multiplication computation cannot be fully used, resulting in inefficiency. On the other hand, existing coarse-grained pruning methods proposed to achieve high computing efficiency are mostly specific to convolutional neural networks (CNNs). These methods mainly prune the unique structures in convolutional layers such as filters [8], whereas they rarely carry coarse-grained pruning on fully connected networks, which are very straightforward and effective models with many connections. Therefore, in order to address these limitations, a coarse-grained pruning method suitable for fully connected structures, which removes model redundancy and improves computing efficiency without greatly harming accuracy, will be the focus of this paper.

For coarse-grained pruning models, there are many sparse matrix compression storage approaches for optimizing the pruned weight matrices operations. Block Sparse Row (BSR) as an effective compression format for sparse matrix-vector multiplication (SpMV) acceleration is very suitable for storing sparse weight matrices with continuous data sub-blocks. We were inspired by the above two facts to design the strategy of block as the coarse-grained pruning unit in this paper. In our method, the weight matrix is divided into square sub-blocks. Whether the sub-block is retained or pruned depends on its importance in the network. After these remaining nonzero elements in the weight matrix are aggregated into blocks with regular structures, the BSR storage format is employed to compress the network and to accelerate the weight matrix computation.

We conducted experiments on the MNIST, Fashion-MNIST, and CIFAR-10 benchmark datasets. It is found that some pruning models maintain accuracy when achieving a large pruning rate. At the same time, the pruned network may still achieve a reasonable performance on the classification effect compared with random sparse networks and some existing pruned models. The relationships between sparsity and accuracies and between pruning granularities and accuracies are also discussed for our method. With the increase in sparsity and pruning granularity, the accuracy has a decreasing trend. It is found that the accuracy is influenced by the sparsity and pruning granularity according to a certain rule.

From the perspective of computation, our block pruning strategy can work well with the BSR storage format. The combination can improve the computational efficiency and cache hit ratio compared with the traditional Compressed Sparse Row (CSR) format.

Our main contributions reported in this paper are as follows:

1. We design a coarse-grained unit called sub-block, which is applicable to the fully connected structure. Based on this structure, we propose a novel block-based division strategy and coarse-grained block pruning method for fully connected structures, which learns the weights and network structure. Accuracies with different sparsity and different pruning granularities are explored. This method can also be combined with CNNs.
2. We exploit the BSR storage format to store the pruned blocky weight matrices, which can effectively accelerate in computation and compress in storage.
3. We show that the cache hit ratio during network inference can be improved when the compressed model is stored by BSR. We analyze the reasons for the high computational efficiency from this aspect.

## 2. Related Works

### 2.1. Pruning Criterions

Many studies have designed different pruning guidelines on selecting the important connections. In [9], Han et al. investigated the criterion based on absolute values. The relatively low absolute values of weights in the network would be set to 0. It cut down the number of parameters by 9–13 times. In [10], Ayinde et al. removed duplicate weights or similar weights based on relative cosine distances. In [11], Wu et al. designed a multi-objective particle swarm optimization algorithm for evolutionary pruning. The objectives of accuracy and the sparsity were iteratively optimized to prune network with remaining accuracy. In [12], Lee et al. designed a saliency criterion based on connection sensitivity. The proposed saliency measurement criterion evaluated the gradient of the loss function with respect to the connections. The network at initialization was pruned using this criterion. The method reduced the network scale with the minimal or no loss of accuracy on AlexNet [13] and VGG [14]. In [15], Lee et al. characterized the initialization condition from the signal propagation perspective and made the connection sensitivity reliable. Then, they pruned the initialized network based on connection sensitivity.

### 2.2. The Granularity of Pruning

#### 2.2.1. Fine-Grained Pruning

Fine-grained pruning is a technique for pruning individual weights. In [6], Han et al. sorted the absolute values of weights in the network and deleted connections below the threshold. It reduced the number of parameters of LeNet-300-100 by 12 times. This method also exceeded the accuracy of the original network. The Lottery Ticket Sparse Network [7] cut down the individual weights and found a sub-network that accounts for 20% of the original LeNet-300-100 network parameters scale, achieving the accuracy of the original network. Boyd et al. [16] pruned networks by using ADMM [17]. The regularization target was updated dynamically in each ADMM iteration. This resulted in high model compression performance.

#### 2.2.2. Coarse-Grained Pruning

Many proposed coarse-grained pruning methods are used to regularize the structures of neural networks. They are usually applied to CNNs. In [8], the sums of absolute values of weights in the CNN’s structures (kernels and filters) were calculated. Then, these coarse-grained structures with low sums were pruned. The sparsity on AlexNet [13] reached 75.2% after pruning. Li et al. [18] significantly reduced the computation cost by removing filters. The Structured Sparsity Learning (SSL) method [19] regularized convolutional network structures (e.g., channels, filters, etc.). The regularization was realized by adding a group Lasso regularization item to the loss function. During the optimization, some regularized structures were zeroed out. For ConvNet [13], each convolutional layer pruned 50%, 70.7%, and 36.1% of the parameters. The SSL method also used group Lasso regularization on fully connected layers. However, the influence of different pruning granularities on accuracies was not considered. Lin et al. [20] pruned filters by integrating two different structured regularizers into the objective function. The pattern-based convolution (PCONV) pruning method [21] pruned specific convolution kernel patterns. In [22], He et al. designed a novel method for filter pruning via the geometric median. This method compressed the models by pruning the redundant convolution kernels rather than the less important kernels. Lin et al. [23] calculated the rank of feature map and sorted them. The filters with low-rank feature maps were pruned. In [24], Chen et al. proposed a channel pruning method. This method assessed the importance of channels by considering the weights in convolutional layers and the scaling factors in the BatchNorm layers.

### 2.3. Sparse Matrix Storage and Computational Optimization

Many studies explored storage methods and computational optimization methods on pruned sparse matrices. For sparse matrix storage formats, Bell et al. [25] summarized a large number of sparse matrix representations and storage approaches, such as coordinate (COO) format, ELLPACK format, CSR format, etc. Yang et al. [26] considered the probability distribution of nonzero elements in the matrix to obtain dense blocks, thereby improving the performance of SpMV. In [27], a sparse matrix partitioning algorithm was proposed based on hybrid sparse matrix storage format, which overcomes the limitation of single sparse format and combines the characteristics of each selected format (ELLPACK format and COO format). The matrices can be partitioned and stored using hybrid format and can then be respectively assigned to the CPU and the GPU for simultaneous calculation, which accelerates the SpMV operations. The SSL method [19] reached 3.1× and 5.1× layer-wise acceleration on the GPU and the CPU in AlexNet [13] by storing the pruned sparse weight matrices in the format of CSR.

## 3. Block Pruning Model

In our method, the block-based division and the coarse-grained block pruning are performed on fully connected structures to regularize the weight matrices firstly. After the model is pruned, the sparse weight matrices with dense blocks are stored in the BSR format. The block-based division structure is specified in Section 3.1, and the block-based pruning approach is introduced in Section 3.2. The compression method is also discussed in Section 3.3.

### 3.1. Block-Based Division Structure

For fully connected networks, the *a* ×*b* weight matrix of each layer is divided into many *n*× *n* square sub-blocks without overlap, where *n* is the division granularity. In other words, the granularity is length of the side of the sub-block.

The upper-left vertex coordinates of each sub-block are $\left(k\times n\;,\;i\times n\right)$, where *k*, *i* = 0, 1, 2⋯and *k* × *n* < *a*, *i* × *n* < *b*. The lower-right vertex coordinates of each sub-block are $\left(\min\left(\left(k+1\right)\times n\;,\;a\right)\;,\;\min\left(\left(i+1\right)\times n\;,\;b\right)\right)$, where *k*, *i* = 0, 1, 2⋯. Under this partition, the entire weight matrix can be regarded as a matrix arranged by a multitude of sub-blocks.

### 3.2. Block-Based Pruning Method

**Figure 1:**
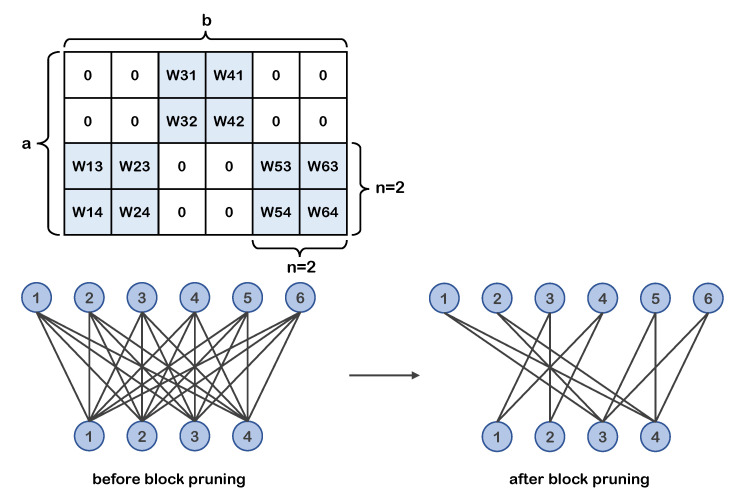
Weight matrix example (**top**) of 2 × 2 block pruning. Corresponding neurons and connections structure example (**bottom**). The retained weights in the weight matrix are distributed in blocks.

Based on the above block division structure, we propose a new block coarse-grained pruning method that outputs a sparse blocky sub-network. This method prunes the divided weight sub-blocks rather than individual weights. The pruning granularity is the same as the division granularity. Figure 1 shows the *a* × *b* weight matrix example after block pruning and the corresponding special pruned neural network structure. In the matrix example, the white areas represent sub-blocks that have been pruned.

Our weight-pruning criterion for sub-blocks is designed on the basis of the low-magnitude weight removal strategy in [6]. In our method, scores of sub-blocks are calculated based on the average of absolute values of weights in sub-blocks. Whether sub-blocks in the weight matrix are pruned or not depends on their scores. The connections in sub-blocks with lower scores are removed from the network. The formulas are as follows:

(1) $$S_{i}=\frac{\sum \left|w_{g_{i}}\right|}{\left|g_{i}\right|}$$

(2) $$R_{i}=\frac{S_{i}}{max\left(S\right)}$$

where $g_{i}$ represents the ith sub-block in the weight matrix and $\left|g_{i}\right|$ is the number of weights in the ith sub-block, which is not identical in the blocks near edges. $W_{g_{i}}$ means the weight values in the sub-block $g_{i}$. $S_{i}$ denotes the average of absolute values of the weights in the ith sub-block. The $\max\left(S\right)$ means the maximum value of all $S_{i}$, and the scores $R_{i}$ are normalized to the range $\left[0,1\right]$. After calculating the scores, the pruning procedure is performed.

We employed the iterative pruning proposed by [6] in the process of model compression. Compared with the one-shot direct pruning, it can make the model sparser while maintaining the accuracy. Specifically, pruning and then retraining is one iteration. In every iteration, sub-blocks are sorted by their scores $R_{i}$ first. Then, a threshold value is calculated according to the pruning rate and the number of the remaining weights (The pruning rate is the ratio of the number of weights to be pruned to the total number of nonzero weights remaining). Finally, the weights $W_{g_{i}}^{\mathrm{pruned}}$ in the sub-blocks with $R_{i}$ less than this threshold are set to 0 and the weights in other sub-blocks retain their original values $W_{g_{i}}$, as shown in the Equation (3).

(3) $$W_{g_{i}}^{pruned}=\left\{\begin{array}{cc} 0 & R_{i}<threshold\\ W_{g_{i}} & R_{i}\geq threshold \end{array}\right.$$

After that, the model is retrained for multiple epochs to fine-tune the remaining weights and to enhance the performance. Let $\left|\mathrm{weight}\right|$ denote the total number of weights. During the back propagation, a mask $m\in {\left\{0,1\right\}}^{\left|\mathrm{weight}\right|}$ can be set based on the corresponding weight and can make a element-wise product with the weight gradient $G_{w}$ to ensure that the removed weight values are not updated.

**Figure 2:**
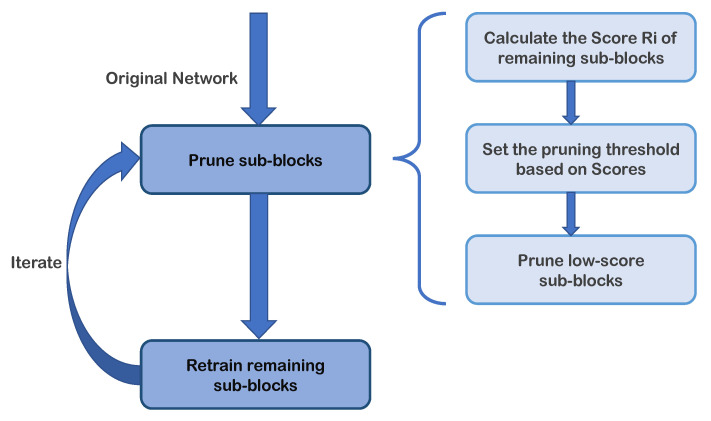
The process diagram of iterative pruning. The pruning-step and retraining-step are performed alternately. The sub-diagram on the right details the flow of the pruning phase in our blocky compression strategy.

To visualize the iterative pruning method, the iterative process is shown in Figure 2. After several such iterations, the unimportant connections are gradually pruned to reach the target sparsity and the well-performed compressed model is obtained in a gradual manner.

The sparse blocky structure produced by this coarse-grained pruning method has better spatial aggregation, which is much more convenient for the following computational optimization work.

### 3.3. Block Sparse Matrix Storage and Computation

The CSR format is a typical sparse matrix storage method that has many advantages [25,28]. The CSR format tends to store nonzero elements in the weight matrix one by one by recording row offsets, values, and column indices rather than the whole matrix. Such information is able to facilitate quick access to data and to benefit compression in storage space.

However, in this paper, pruned sparse weight matrices are stored in the BSR format. BSR is similar to the CSR format, except that the elements are stored and recorded block by block. After block-based pruning, there are many compact weight blocks in the weight matrices so BSR format is more effective in storage and computation.

## 4. Experiments and Results

We conducted block-based pruning experiments on the LeNet-300-100 [29] model and on the fully connected layers of the ResNet18 [30] model using three datasets—MNIST, Fashion-MNIST, and CIFAR-10—to examine the effectiveness of our block pruning method. The classification accuracies were evaluated, and the relationships between accuracies and sparsity and between accuracies and pruning granularities were explored. The computation efficiency experiments were then carried out on the blocky sparse matrices. Furthermore, we performed the experimental analysis about the cache hit ratio. In the end, we combined this block pruning strategy with the convolutional network and then evaluated the accuracy.

### 4.1. Block-Based Pruning Strategy on Fully Connected Network

#### 4.1.1. Accuracy Evaluation Experiments

We performed coarse-grained block-based pruning iteratively on a classic LeNet-300-100 fully connected network, which is a three-layer network. This network is a very popular fully connected network. This facilitates comparisons with other classic methods.

During iterative pruning, for the first two layers the pruning rate is set to 0.2, that is, 20% of remaining parameters were pruned each time. In the output layer, since the number of parameters takes a very small proportion in the whole number of parameters (1000 in 266,000), the pruning rate is set to 0.1, which is the half of the pruning rate for the first two layers. Such a pruning rate setting learned from [7,31] is commonly used.

In the experiments, we applied this block pruning strategy on the two datasets MNIST and Fashion-MNIST to evaluate the results.

**MNIST.** The MNIST handwritten digital dataset, which contains 60,000 samples for training and 10,000 samples for testing, was adopted. MNIST is a typical and popular dataset that is suitable for fully connected networks.

**Figure 3:**
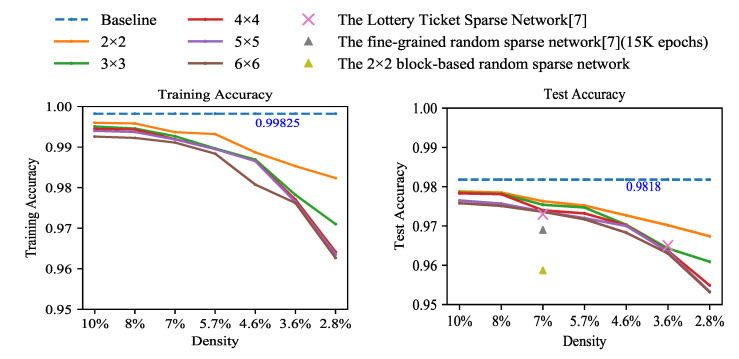
The accuracy for training and testing of LeNet-300-100 on the dataset of MNIST. Solid lines are our block coarse-grained pruned networks.

Experiments were conducted to explore accuracies with different sparsities and different pruning granularities. One of our focuses is how sparsity and pruning granularity affect accuracy. The baseline is the unpruned LeNet-300-100 fully connected network trained on the MNIST dataset. We performed iterative block pruning on the baseline model. The experimental results are shown in Figure 3. Each solid line with different colors represents a pruning granularity. The vertical axis represents accuracy. The horizontal axis marks density formed during the iterative pruning process. (The density refers to the percentage of unpruned weights in the weight matrix to the total number of weights. The relationship between density and sparsity is density = 100% − sparsity. For a more intuitive representation of the percentage of remaining parameters in the experiments, we chose the density as the horizontal axis.) During the experiments, the model at each density was obtained by pruning the model at the previous density and retraining. Each model with different sparsities and pruning granularities was trained for 1000 epochs. The cross-entropy loss function was used for network training. In the experiments, it is found that, when the density is more than 8%, the accuracy of each model is almost the same and the difference in accuracy is not obvious so models with a density less than 8% are the main focus.

When the density is 8%, each model can still maintain a similar accuracy to the baseline. It can be observed from Figure 3 that there is a downward trend for accuracy with the increase in sparsity. When the number of weights is less than a threshold, almost every weight is useful so over-pruning results in a loss of accuracy.

We also find that, with the same sparsity, as the pruning granularity increases, accuracies also decrease. It is caused by the limitation in search space due to the coarse-grained pruning rule. For example, test accuracy decreases from 97.85% to 97.51% (pruning granularity is from 2 × 2 to 6 × 6) when the density is 8%. When the density is 2.8%, the gap tends to be relatively obvious with the increase in pruning granularity, as shown in Table 1.

To validate our pruning method, the comparison between our coarse-grained pruning network and the random pruning networks is made. Two kinds of random sparse networks are investigated. One is the fine-grained random pruning sparse network, trained about 15 K epochs in [7]. The other is the block-based random pruning network with the same 1000 training epochs as our weight magnitude-based blocky pruning method for better comparison. The accuracies of both random networks are the average of 10 trials. The result of the fine-grained random sampling sparse network is from [7]. From Figure 3, when the density is 7%, our method achieves much better performances than the fine-grained random sparse network and block-based random sparse network, which demonstrates the effectiveness of our pruning method.

In addition, our model is compared with the fine-grained pruned model of The Lottery Ticket Sparse Network, the accuracy of which is obtained from [7]. The pruning criterion of [7] is also based on the weight magnitude. After training for 1000 epochs in both cases, it is found that the accuracies of some of our block-based pruned models are higher than those of [7] when the densities are 7% and 3.6%. The little advantage is most likely caused by different training methods rather than the model properties. Our training process continues from the remaining weights after pruning but their model restarts with initial random weights.

We also compared our block pruning method with methods that employed other pruning criterions. Reference [11] exploited the multi-objective particle swarm optimization algorithm for pruning, which consists of two objective terms of the accuracy and the sparsity. The comparison results are shown in the Table 2. Our block pruning model performed better than [11] with less parameters.

**Figure 4:**
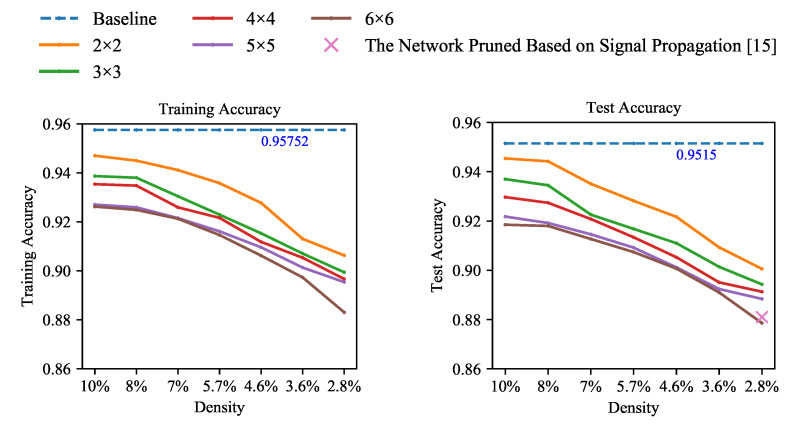
The accuracy for training and testing of LeNet-300-100 on the dataset of Fashion-MNIST. Solid lines are our block coarse-grained pruned networks.

**Fashion-MNIST.** To verify the generalizability of the findings, we also conducted block pruning experiments on this dataset of Fashion-MNIST. The Fashion-MNIST dataset describes clothing classification images. It consists of 60,000 training images and 10,000 test images. We performed pruning experiments on Fashion-MNIST using similar experimental settings to that of MNIST. Every model with different sparsities and pruning granularities trained 1000 epochs. The results are shown in Figure 4.

From the experimental results, the accuracy variation law shown on the Fashion-MNIST dataset is the same as that of the MNIST dataset. It can fully reflect the relationships between accuracies and sparsity and between accuracies and pruning granularities. The accuracies decrease with the increase in sparsity and pruning granularity.

We also conducted comparative experiments on this dataset of Fashion-MNIST. The sparse network model proposed by [15] adopted another pruning strategy. In [15], the pruning criterion were designed based on the signal propagation perspective. From Figure 4, our blocky pruned model reaches a higher accuracy than [15] with the same density. It demonstrates that our block pruning method performs well in terms of preserving accuracy.

In our experiments, the accuracy has a slight gap compared with that of the baseline, which is acceptable. One of the reasons is that the number of training epochs is a little small. On the other hand, our target is analyzing the relationships between sparsity and accuracies and between pruning granularities and accuracies and obtaining the sparse weight matrix structure suitable for computation rather than striving for excellent accuracy.

It is also worth mentioning why fully connected structures are mainly considered in the experiment rather than CNNs. On the one hand, the concept of a coarse granularity applicable to fully connected structures is currently unclear. Therefore, we designed sub-blocks as the coarse-grained pruning units and conducted block pruning on fully connected structures. On the other hand, CNNs share weights and reduce the number of parameters. However, floating-point operations (FLOPs) are not reduced by much. Taking MNIST dataset as an example, without greatly increasing error rate, FLOPs and the number of weights of our pruned blocky network are significantly less than those of LeNet-5, as shown in Table 3. Therefore, we believe that it makes sense to only perform block-based coarse-grained pruning on fully connected structures.

#### 4.1.2. Block Sparse Matrix Computation and Cache Hit Ratio Experiment

In this section, we intend to explore the performance of our pruning strategy from the perspective of computational efficiency and cache hit ratio. We first conducted weight matrix-vector multiplication computation experiments during forward propagation on the MNIST dataset.

The sparse matrices obtained from Section 4.1.1 were used in this computational experiment on CPU. Weights are floating point numbers. In the experiments, the CPU version was i7-6500U and the performance analysis function provided by Intel Math Kernel Library (Intel MKL) was exploited to sample the time span. There are some reasons why we did not carry out experiments on an Nvidia GPU. First, related functions of the cuSparse library [32] split BSR blocks with different sizes, which causes performance jitter. The detailed function implementation is not documented, so we cannot analyze the cause accordingly. Second, though we attempted to design it, the performance of our own CUDA implementation is not as good as cuSparse so it is not persuasive enough to make the comparison. Finally, in our opinion, the CPU results are enough to display the pattern of memory access which is a key point in sparse matrix algorithms.

**Figure 5:**
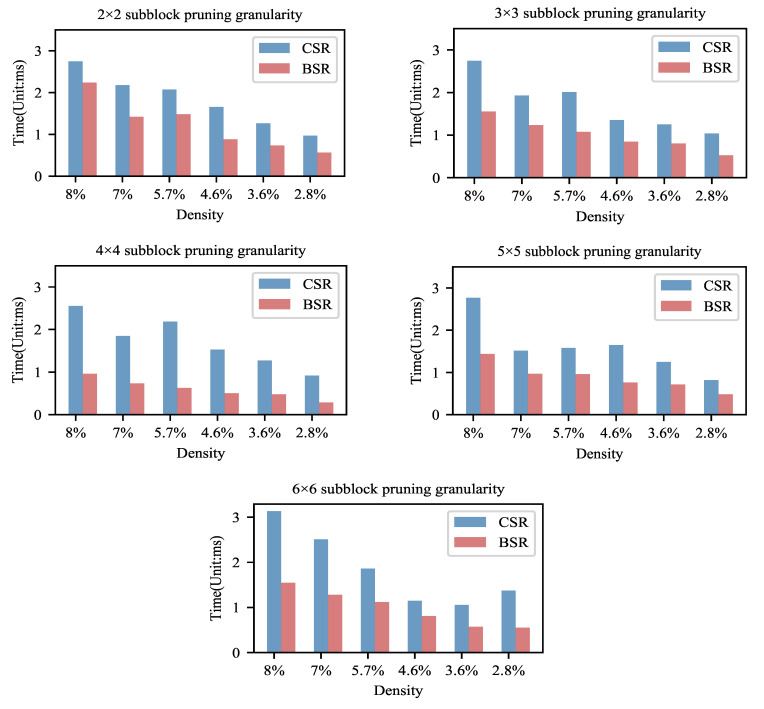
The inference computation time of block sparse models. Note that the computational time of Block Sparse Row(BSR) storage is much less than that of Compressed Sparse Row(CSR). This indicates that it is effective and reasonable to use the BSR format to store a block sparse matrix.

In the experiment, we explored the computational efficiency when our block pruning method was combined with the BSR format. The computational time of sparse matrices stored by the BSR format and the CSR format is also made a comparison. The model settings and the operating environment are the same, for fairness. To avoid cold starting and the random noise effect, the sparse matrix-vector multiplication was iterated 100 times and then the total time was recorded. The experiment was repeated four times for averaging. The experimental results are shown in Figure 5 and Table 4.

From Figure 5, we could observe that, as the density decreases, the computation time of the BSR storage format tends to decrease significantly. This indicates that, as the sparsity increases, the computational efficiency of the BSR improves. We can further analyze the tradeoff between accuracy and computational efficiency based on this variation law. From Section 4.1.1, it can be found that there is no significant loss in accuracy at a density of about 8–10%. Therefore, according to this result, we believe that the model can be block pruned to this extent if the balance between accuracy and efficiency is pursued. At a density below 8%, the accuracy tends to decrease with the increase in sparsity and pruning granularity. However, the computational efficiency of the BSR format can be improved significantly with the increase in the sparsity. We believe that the model of high sparsity and high pruning granularity could be chosen if the accuracy requirement is not strict and the computation and storage resources are limited.

From Table 4, the speedups achieved 1.229×– 3.476× by comparing the BSR storage format with the CSR storage format. All of the pruned models stored in the format of BSR achieved varying degrees of speedups. It can be seen that the combination of this block-based pruning strategy and the BSR storage format can greatly increase the sparse matrix operation speed and can optimize computation.

**Figure 6:**
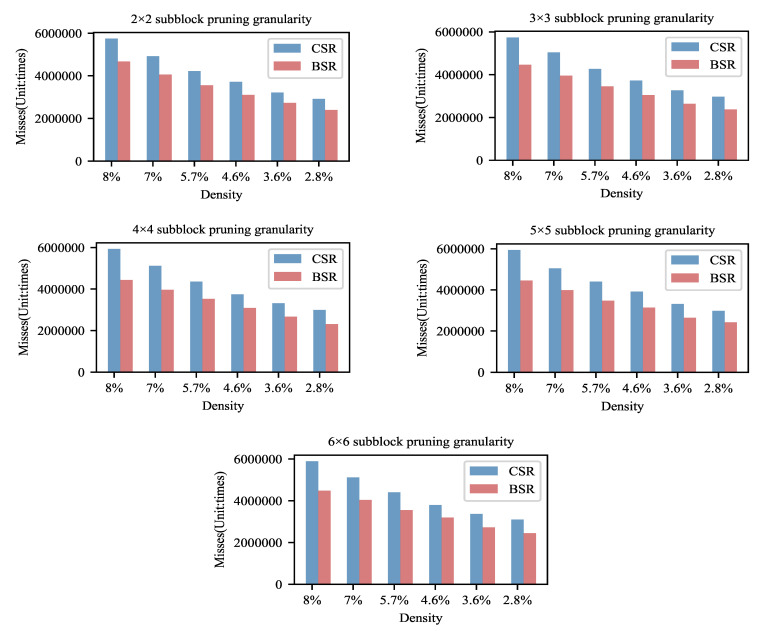
Comparison of the number of misses of L1 cache. The BSR storage format has an advantage in terms of the cache hit rate.

We assume that one of the main reasons for the speedups of the BSR storage format is the higher cache hit ratio. Therefore, the number of misses of L1 cache are measured for various sparsity and pruning granularity models stored in the BSR and CSR formats in the experiments, as shown in Figure 6. All results are the sum of 1000 times network inferences. The number of misses is the average of five experimental results.

From Figure 6, as the density decreases, the number of cache misses is reduced. This variation trend is the same as that of computation time. It reflects the increase in cache hit ratio being one of reasons for the enhancement in the computational efficiency.

It also can be seen that the cache misses using the BSR storage format are, on average, 19.98% less than that using the CSR storage format. Since the data are stored by block in the format of BSR, the BSR approach makes better use of the aggregation of data in terms of the storage mechanism compared with the CSR storage format. The integrity of data access is much more enhanced, so the number of cache updates is reduced and then the cache hit ratio increases in the format of BSR.

For the dataset Fashion-MNIST, we also conducted computational and cache hit ratio experiments. The performance is very similar to that of the MNIST dataset since the size, the density, the division granularities of weight matrices, and the other settings are the same as those of the MNIST dataset. This illustrated that our block pruning strategy is very computationally friendly.

### 4.2. Block-Based Pruning Strategy on Convolutional Network

Though our pruning method mainly focused on the fully connected structures rather than on convolutional structures, the block pruning strategy can combine well with CNNs. In the experiments, we conducted iterative block pruning on the fully connected layers of ResNet18 on the CIFAR-10 dataset. The images in the CIFAR-10 dataset are the natural images in RGB colors.

We added an extra fully connected layer with 300 neurons on top of the original fully connected layer of ResNet18. The unpruned ResNet18 model was trained until convergence with 300 epochs on CIFAR-10 as the baseline model. To focus on the pruning properties of the fully connected layers, the weights in the convolutional layers were fixed during the iterative pruning and fine-tuning. The block pruning was only performed on fully connected layers using the pruning granularity of 2 × 2. The model trained 300 epochs at every iteration. Finally, we cut off 97% of the parameters of the fully connected layers. From the experimental results, the test accuracy improved from 90.37% (baseline model) to 90.73%. This boost demonstrated that our block pruning method found the proper capacity of the network. It can eliminate the redundance and can alleviate overfitting.

## 5. Conclusions and Discussion

This study presented a special block-based division method and coarse-grained block pruning method for fully connected structures. This pruning method can also be integrated into convolutional networks well. The experimental results on the MNIST, Fashion-MNIST, and CIFAR-10 testing datasets show that some models maintain similar accuracies when reaching a large compression rate. Our block-based models do not decrease the classification accuracy compared with the existing fine-grained pruning method and other methods based on different pruning criterions. The correlations between accuracies and sparsity and between accuracies and pruning granularities were also evaluated. The increase in sparsity and pruning granularity has a negative effect on accuracy. The tradeoff between accuracy and efficiency was discussed.

In terms of calculation, all compressed models stored in the format of BSR improved in computation speed compared with the CSR format. The speedups reached 1.229×–3.476×. The CPU cache hit ratio was 19.98% higher than that of the CSR storage format, on average. The experimental results show that our block-based pruning models stored in the format of BSR can ensure the continuity of data access, can improve the cache hit ratio, and can increase the calculation efficiency.

In the future, we intend to design a strategy combining block-growth and block-pruning, which adjusts the network structure more flexibly to give “silent” connections a chance to be reactivated. This algorithm may enhance the accuracy further. In addition, we plan to consider how to fully take advantage of this block sparse structure to significantly reduce the FLOPs of CNNs. For a more accurate computational performance analysis, we will consider the use of Field Programmable Gate Arrays (FPGAs) to implement a special computational model.

## Figures and Tables

**Table 1:** The specific variation in test accuracy on the dataset of MNIST.

	Density	8%	2.8%
Granularity			
2 × 2		97.85%	96.74%
6 × 6		97.51%	95.32%

**Table 2:** Comparison of the accuracies between our block pruning model and the model of [11] on the MNIST dataset. Our model adopted the pruning granularity of 2 × 2.

Model	Method	Error %	Remaining Weights %
	multi-objective optimization algorithm [11]	2.2%	16.64%
LeNet-300-100	**our method**	**1.97%**	13.52%
	**our method**	2.15%	**8%**

**Table 3:** Comparison of floating-point operations(FLOPs) and weights between the convolutional network LeNet-5 and the pruned network LeNet-300-100 with 8% density on the MNIST dataset.

Network	Weights	FLOPs	FLOPs%	Error
LeNet-5 unpruned	431 K	4586 K	100%	0.8%
LeNet-300-100 unpruned	266 K	532 K	11.6%	1.82%
**LeNet-300-100 block pruned (Ours)**	**23 K**	**46 K**	**1%**	**2.15%**

**Table 4:** The sparse matrix computation speedups (The times of the computation speed in the BSR format to that in the CSR format) of block pruned models using the BSR format (compared with the CSR format).

Model		Density	8%	7%	5.7%	4.6%	3.6%	2.8%
	Granularity							
	2 × 2		1.229×	1.534×	1.400×	1.883×	1.722×	1.717×
	3 × 3		1.767×	1.561×	1.864×	1.597×	1.560×	1.971×
LeNet-300-	4 × 4		2.657×	2.509×	3.476×	3.015×	2.663×	3.220×
100	5 × 5		1.931×	1.559×	1.642×	2.161×	1.746×	1.691×
	6 × 6		2.024×	1.962×	1.660×	1.421×	1.854×	2.484×

## Data Availability

Our source codes can be found at https://github.com/ZJia77/BlockPruning.

## Nomenclature

BSR	Block Sparse Row
CSR	Compressed Sparse Row
CNN	Convolutional Neural Network
SpMV	Sparse Matrix-Vector Multiplication
ADMM	Alternating Direction Method of Multipliers
SSL	Structured Sparsity Learning
PCONV	Pattern-based Convolution
COO	Coordinate
FLOPs	Floating-Point Operations
FPGA	Field Programmable Gate Array
